# Antithrombin deficiency caused by *SERPINC1* gene mutation in white matter lesions: A case report

**DOI:** 10.1097/MD.0000000000037721

**Published:** 2024-04-05

**Authors:** Song Wang, Runcheng He, Jian Xia, Wenping Gu, Jing Li, Huan Yang, Qing Huang

**Affiliations:** a Department of Neurology, Xiangya Hospital, Central South University, Changsha, People’s Republic of China; b Clinical Research Center for Cerebrovascular Disease of Hunan Province, Xiangya Hospital, Central South University, Changsha, People’s Republic of China; c National Clinical Research Center for Geriatric Disorders, Xiangya Hospital, Central South University, Changsha, People’s Republic of China.

**Keywords:** antithrombin deficiency, hypercoagulable state, multiple sclerosis, *SERPINC1* gene mutation, white matter lesions

## Abstract

**Rationale:**

White matter lesions (WMLs) are structural changes in the brain that manifest as demyelination in the central nervous system pathologically. Vasogenic WMLs are the most prevalent type, primarily associated with advanced age and cerebrovascular risk factors. Conversely, immunogenic WMLs, typified by multiple sclerosis (MS), are more frequently observed in younger patients. It is crucial to distinguish between these 2 etiologies. Furthermore, in cases where multiple individuals exhibit WMLs within 1 family, genetic testing may offer a significant diagnostic perspective.

**Patient concerns:**

A 25-year-old male presented to the Department of Neurology with recurrent headaches. He was healthy previously and the neurological examination was negative. Brain magnetic resonance imaging (MRI) showed widespread white matter hyperintensity lesions surrounding the ventricles and subcortical regions on T2-weighted and T2 fluid-attenuated inversion recovery images, mimicking immunogenic disease—MS.

**Diagnoses:**

The patient was diagnosed with a patent foramen ovale, which could explain his headache syndrome. Genetic testing unveiled a previously unidentified missense mutation in the *SERPINC1* gene in the patient and his father. The specific abnormal laboratory finding was a reduction in antithrombin III activity, and the decrease may serve as the underlying cause for the presence of multiple intracranial WMLs observed in both the patient and his father.

**Interventions:**

The patient received percutaneous patent foramen ovale closure surgery and took antiplatelet drug recommended by cardiologists and was followed up for 1 month and 6 months after operation.

**Outcomes:**

While the lesions on MRI remain unchanging during follow-up, the patient reported a significant relief in headaches compared to the initial presentation.

**Lessons:**

This case introduces a novel perspective on the etiology of cerebral WMLs, suggesting that hereditary antithrombin deficiency (ATD) could contribute to altered blood composition and may serve as an underlying cause in certain individuals with asymptomatic WMLs.

## 1. Introduction

White matter lesions (WMLs) mainly refer to hyperintensity lesions shown on T2-weighted and T2 fluid-attenuated inversion recovery images, also known as white matter hyperintensities.^[1]^ There are differences in the etiological classification of WMLs in patients of different ages: middle-aged and elderly people with cerebrovascular disease-related risk factors are first considered to be related to cerebral small vessel disease,^[2]^ symmetrical WMLs in children require performing genetic testing to identify whether they are inherited metabolic leukoencephalopathy,^[3]^ and the most common cause of WMLs in youngsters is chronic inflammatory demyelinating lesions led by multiple sclerosis (MS).^[4]^

Previous studies have declared that vasogenic WMLs were associated with migraine, cerebrovascular risk factors and cerebral ischemic damage, but the specific pathophysiological mechanism has remained unclarified.^[1,2]^ In this paper, we report a young man who presented with headache, whose WMLs mimicking immunogenic disease—MS. The genetic testing revealed a novel *SERPINC1* missense mutation, which may be the underlying cause of multiple intracranial WMLs. This case provides a new perspective for the differential diagnosis of MS, widens the etiological classification of vasogenic WMLs, and calls for discussions of the new mechanism of WMLs.

## 2. Case presentation

A 25-year-old male presented to the neurology department with recurrent headaches for more than 4 years. His headache was characterized by bilateral paroxysmal cephalalgia, mostly occurring in the afternoon, lasting 1 to 2 hours, which can be relieved spontaneously after rest. Limb numbness sometimes occurs when the patient gets emotional. The patient was healthy previously with no smoking or alcohol habits, and the neurological examination was negative.

**Figure 1.**
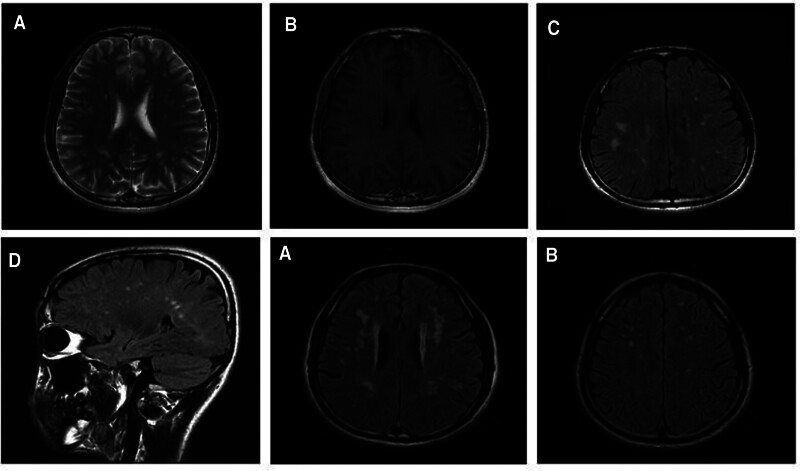
Brain MRI images of the patient (A–D) and his father a, b. Axial T2-weighted (A), T2 fluid-attenuated inversion recovery (C) and Sagittal T2 fluid-attenuated inversion recovery (D) images showed several discrete abnormal hyperintensity lesions in the periventricular deep white matter as well as frontal and parietal lobes of both cerebral hemi-spheres, most lesions were distributed perpendicular to the lateral ventricles, and some became confluent; T1-weighted contrast (B) images showed lesions were not enhanced. Axial T2 fluid-attenuated inversion recovery a, b. Images of the father showed more bilateral deep paraventricular white matter confluent lesions and more obvious brain atrophy. MRI = magnetic resonance imaging.

Brain magnetic resonance imaging (MRI) showed multiple white matter hyperintensity lesions on T2-weighted and T2 fluid-attenuated inversion recovery images (Fig. 1A–D). The patient was initially diagnosed as MS due to the features of intracranial lesions at MRI, the young age of onset, and the occasional limb numbness. However, his serum and cerebrospinal fluid examination did not demonstrate oligoclonal bands, while the cerebrospinal fluid IgG index was normal either.

**Figure 2.**
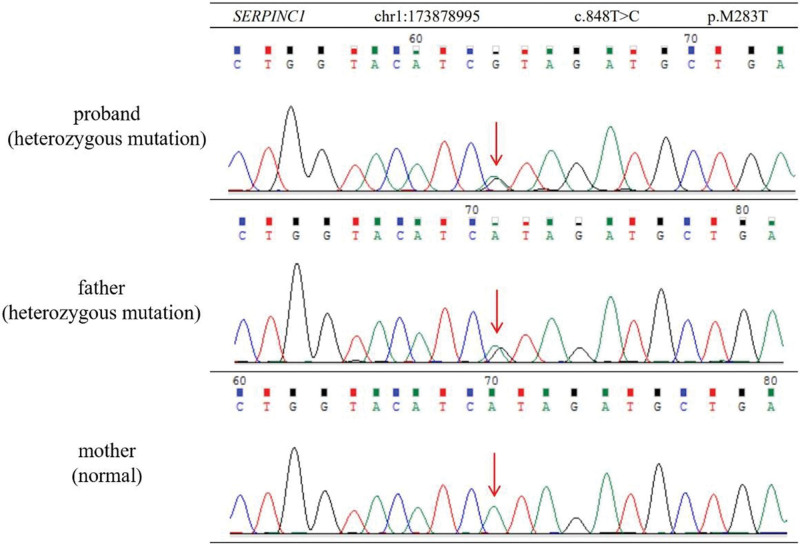
Sanger sequencing showed a novel missense mutation of *SERPINC1 (SERPINC1*: NM_000488.3; c.848T > C: p.M283T). Red arrows indicate position of the mutations.

Furthermore, since the patient was a youngster and had few vascular risk factors, we needed to exclude hereditary leukoencephalopathy. We performed whole exon sequencing on the patient and detected a novel heterozygous missense mutation in *SERPINC1* gene (1q25.1) in the proband (Fig. 2), which is related to antithrombin deficiency (ATD). The sanger sequencing results of his parents revealed the *SERPINC1* gene mutation in the father (Fig. 2). It was predicted to be likely pathogenic by Mutpred2 software (Table 1).

**Table 1:** Pathogenicity prediction of the mutation.

AA change	Mutation taster	PROVEAN	Mutpred2	phyloP
P.M283T	Disease causing	Deleterious	0.841	Conserved

*Note: in Mutpred2, score threshold of 0.5 would suggest pathogenicity.

Consistent with genetic testing, AT III activity in this patient was slightly decreased (72.2%, reference range: 80%–120%), while AT III antigen level were within the normal range (235 mg/L, reference range: 180–392 mg/L). Similarly, thrombophilia-related texts showed lower AT III activity (75.1%) and normal AT III antigen level in the father (263.3 mg/L), and MRI showed multiple asymptomatic WMLs (Fig. 1a, b).

It is also worth mentioning that according to the results of transcranial doppler and transthoracic echocardiography, the patient was diagnosed as patent foramen ovale, which is the most likely reason for his migraine. While his father did not have any neurological clinical symptoms such as headache or limb numbness, and transthoracic echocardiography showed negative signs.

The patient received percutaneous patent foramen ovale closure surgery and took oral antiplatelet recommended by cardiologists and was followed up for 1 month and 6 months after operation. While the lesions on MRI remain unchanging, the patient described that the pain degree, attack frequency and duration of headache were significantly improved compared with before.

## 3. Discussion

In this case, the patient was initially misdiagnosed as MS because he lacked risk factors for cerebrovascular disease and his age was consistent with the demographic features of MS; more importantly, the patient irregular oval lesion on MRI mimicked MS.^[5]^ However, the patient’s cerebrospinal fluid autoimmune examination showed negative, although the lesions involved the paracortical and paraventricular white matter, they did not involve the infratentorial region with abundant blood supply; and the lesions were not enhanced when the patient’s migraine aggravated, which could not reflect the dis-semination of lesions in space and in time of MS. In addition, the symptoms of paroxysmal headache are uncommon in MS patients.

The heterozygous mutations of *SERPINC1*: NM000488.3; c.848T > C: p.M283T detected in the proband and his father were associated with ATD. ATD refers to patients whose AT activity levels are consistently below 80% (or the lower limit of the reference range of the test) and can be divided into acquired ATD and hereditary ATD.^[6]^ Hereditary ATD is mostly caused by mutations in the coding gene of AT—*SERPINC1*, till now, hundreds of *SERPINC1* mutations have been identified. Hereditary ATD is divided into 2 clinical phenotypes: type I: decreased AT activity and AT antigen level; and type II: decreased AT activity levels (i.e., defective AT function) but normal AT antigen level.^[7]^ The patient and his father are diagnosed with type II hereditary ATD.

After searching the literature, we discovered that the missense mutation of *SERPINC1*: NM_000488.3; c.848T > C has never been reported before and it is the first study of WMLs correlated to *SERPINC1* mutations. The exact mechanism WMLs caused by *SERPINC1* mutation remains unclear yet. At the very beginning, we advocated the paradoxical embolism hypothesis: in fact, subcortical and periventricular WMLs are common in many children and young adults, which are considered to be associated with patent foramen ovale.^[8]^ In this case, the anticoagulation mechanism of the patient is defective, there could be microembolization of small vessels and cryptogenic stroke if emboli move across the patent foramen ovale form small peripheral veins into the arterial circulation.^[9]^ However, since we found out that the patient’s father also had many intracranial WMLs without patent foramen ovale, we preferred the hypercoagulable state hypothesis: hereditary thrombophilia, represented by ATD, could increase the risk of microvascular thrombotic diseases and the susceptibility to atherosclerosis.^[10]^

## 4. Conclusion

The multiple WMLs shared by this patient and his father suggest that ATD caused by the *SERPINC1* gene mutation maybe a potential cause of abnormal WMLs. This report identifies a novel missense mutation that expands the clinical phenotype of *SERPINC1* and provides a novel perspective on the etiological classification of WMLs as well as the differential diagnosis of MS.

## Acknowledgments

The authors are thankful to the patient and his family members for their participation in this study.

## Author contributions

**Conceptualization:** Qing Huang.

**Data curation:** Jing Li, Huan Yang.

**Funding acquisition:** Qing Huang.

**Supervision:** Jian Xia, Wenping Gu.

**Writing – original draft:** Song Wang.

**Writing – review & editing:** Song Wang, Runcheng He, Qing Huang.
